# Mid-term outcomes of the modified Dunn procedure for slipped capital femoral epiphysis: results from a north African pediatric hip unit

**DOI:** 10.1186/s13018-024-05369-5

**Published:** 2025-01-07

**Authors:** Mohammad Kamal Abdelnasser, Ahmed Abdelazim Hassan, Mohammed Ibrahim, Abdelkhalek Hafez Ibrahim, Nariman Abol Oyoun

**Affiliations:** 1 Department of Orthopaedics and Trauma Surgery Faculty of Medicine, Assiut University Hospitals, Assiut, 71515 Egypt; 2 Centre for Spinal Studies and Surgery, Queen`s Medical Centre, Nottingham University Hospitals NHS Trust, Nottingham, UK

**Keywords:** SCFE, Modified dunn procedure, AVN, Unstable SCFE

## Abstract

**Background:**

Slipped Capital Femoral Epiphysis (SCFE) is a prevalent pediatric orthopedic condition. Treatment options range from in situ pinning to various osteotomies, with the Modified Dunn procedure gaining significant attention over the past two decades. However, the suitability of this procedure for different SCFE subtypes and the risk of avascular necrosis (AVN), particularly in moderate and severe cases, remains controversial. This study aims to report the midterm clinical and radiographic outcomes of the Modified Dunn procedure in treating SCFE, emphasizing the factors contributing to AVN development.

**Patients and methods:**

We conducted a prospective case series between 2014 and 2022, enrolling patients with moderate and severe SCFE who were treated using the Modified Dunn procedure by a single experienced hip surgeon. Patients were followed up clinically and radiologically for a minimum of two years.

**Results:**

Thirty-six patients (29 males, 7 females) with moderate and severe SCFE were included, with an average age of 14 years and a mean follow-up of 49.28 months (range: 24–118 months). Statistically significant improvements were observed in clinical and radiographic parameters at the final follow-up compared to pre-operative data. Five patients developed AVN, though no specific risk factor reached statistical significance regarding AVN development. While most AVN cases occurred early in the learning curve, this trend was not statistically significant.

**Conclusion:**

Modified Dunn is a safe and effective option for treating moderate to severe SCFE, offering superior femoral head realignment and patient-reported outcomes. When performed by experienced surgeons, it results in acceptable complication rates, including AVN.

**Level of evidence:**

Level IV Prospective Case series.

## Introduction

Slipped Capital femoral epiphysis (SCFE) is adolescents’ most common hip disorder [1]. While mild SCFE with a slip angle of less than 30° can be effectively managed with in situ fixation, the optimal treatment for moderate and severe SCFE remains debated [2]. Historically, attempts to improve capital alignment through open osteotomy procedures [3, 4] have been associated with high rates of avascular necrosis (AVN).

Leunig et al. [5] introduced a modification to Dunn’s procedure, which has since shown favorable outcomes with low AVN rates in some studies. However, other studies have reported a high risk of AVN and complications, questioning the procedure’s reproducibility. Additionally, most studies have not provided detailed AVN rates concerning SCFE subtypes in terms of stability and chronicity.

The primary outcome of this study is to report the midterm clinical and radiological outcome of the modified Dun procedure for SCFE, shedding more light on its reproducibility. The secondary outcome is to study the effect of different disease-related variables on the incidence of AVN.

## Patients and methods

**Fig. 1:**
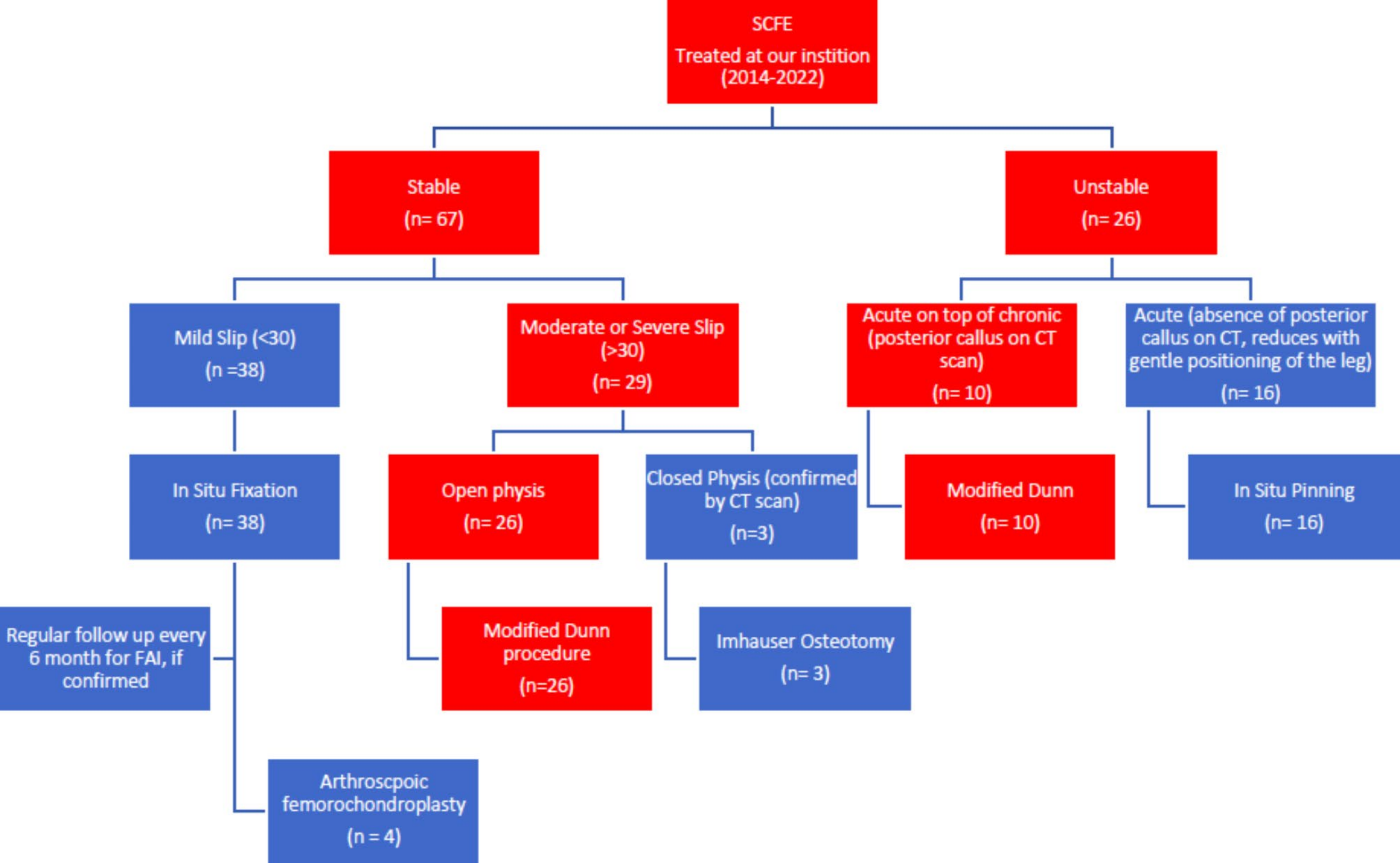
Patient flowchart with our algorithm for management of SCFE

All cases of moderate and severe SCFE with an open physis presented to our department between 2014 and 2022 were consecutively included in this prospective case series. Our algorithm for management of SCFE cases is shown in Fig. 1. All moderate and severe stable slips with an open physis (26 in number) as well as acute on top of chronic unstable slips (10 in number) were treated prospectively by a modified Dunn procedure through safe surgical dislocation. Mild stable slips and acute slips without evidence of posterior callus on CT were fixed in situ (with gentle positioning of the limb in acute ones) and followed up 6-monthly for clinical evidence of femoroacetabular impingement (FAI), where arthroscopic osteochondroplasty was performed. Patients with closed physes were diverted to an extracapsular realignment procedure (Imhäuser osteotomy). Patients with established osteonecrosis or previously operated hips were excluded. The study was approved by the institutional review board, and informed written consent was obtained from the patients’ legal guardians.

All surgeries were performed by the first author adhering to the original Bernese technique described [5]. Gibson’s approach was used in all cases [6]. After doing the tri-gastric trochanteric osteotomy and capsulotomy, the epiphyseal-metaphyseal junction was assessed, and if there was any doubt about physeal stability, provisional fixation with 2 mm threaded K-wires was done. After dislocation, epiphyseal vascularity was assessed by drilling using a 2 mm smooth K- wire in the non-weight bearing area of the epiphysis [7].

**Fig. 2:**
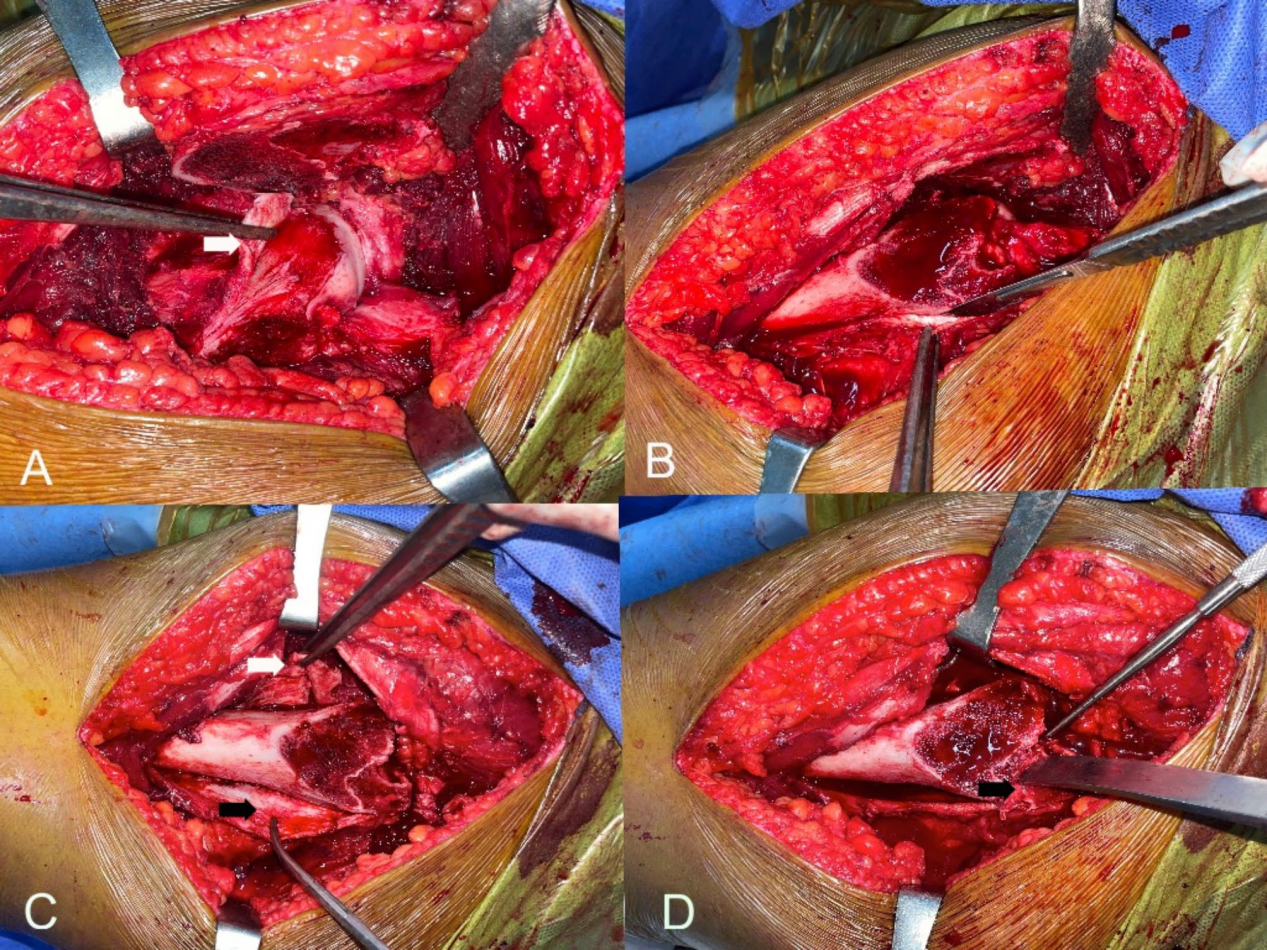
**A**) L-shaped anterior retinacular incision (white arrow) in stable SCFE. **B**) Subperiosteal dissection with a scalpel was made for the posterolateral retinacular flap. **C**) Anterior (white arrow) and posterior retinacular (black arrow) flaps. **D**) To complete the separation of the posterolateral flap, the small remaining bony chip of the posterolateral stable trochanter (Black arrow) was separated using a straight osteotome through the cut surface of trochanter

Any damage to the labrum or acetabular cartilage was also documented. To create retinacular flaps, the head was relocated, and then a longitudinal retinacular incision was made along the femoral neck anterior to the retinacular vessels (Fig. 2). In case of stable SCFE where the retinaculum is stretched and not torn, an additional transverse incision was done anteriorly at the epi-metaphyseal junction away from the retinacular vessels to make an L- shaped incision [8]. Apophyseal osteotomy was done in 5 cases. However, the apophyseal fragment was usually large, which made reduction at the end difficult and may need additional screw fixation. Instead, we continued subperiosteal dissection of the posterior retinaculum using a scalpel, helped with small periosteal elevator on the posterior aspect of the neck proximally and on the posterior aspect of the femur distally gradually with internal rotation of the hip to facilitate the exposure, till a small part of the posterolateral stable trochanter proximal and dorsal to the apophyseal line to which the retinaculum with external rotators is still attached remained. This bony chip was separated using a straight osteotome through the cut surface of the trochanter till reaching the medial cortex, which was broken by leveraging the osteotome in a posterior direction. This is one slight modification of the original described technique of apophyseal osteotomy. After completion of the posterior retinacular flap, the hip was then redisclosed, and subperiosteal dissection was made for the anterior flap. The threaded wires fixing the epiphysis were then removed. The epiphysis was manually stabilized to avoid accidental tension on the retinacular vessels.

**Fig. 3:**
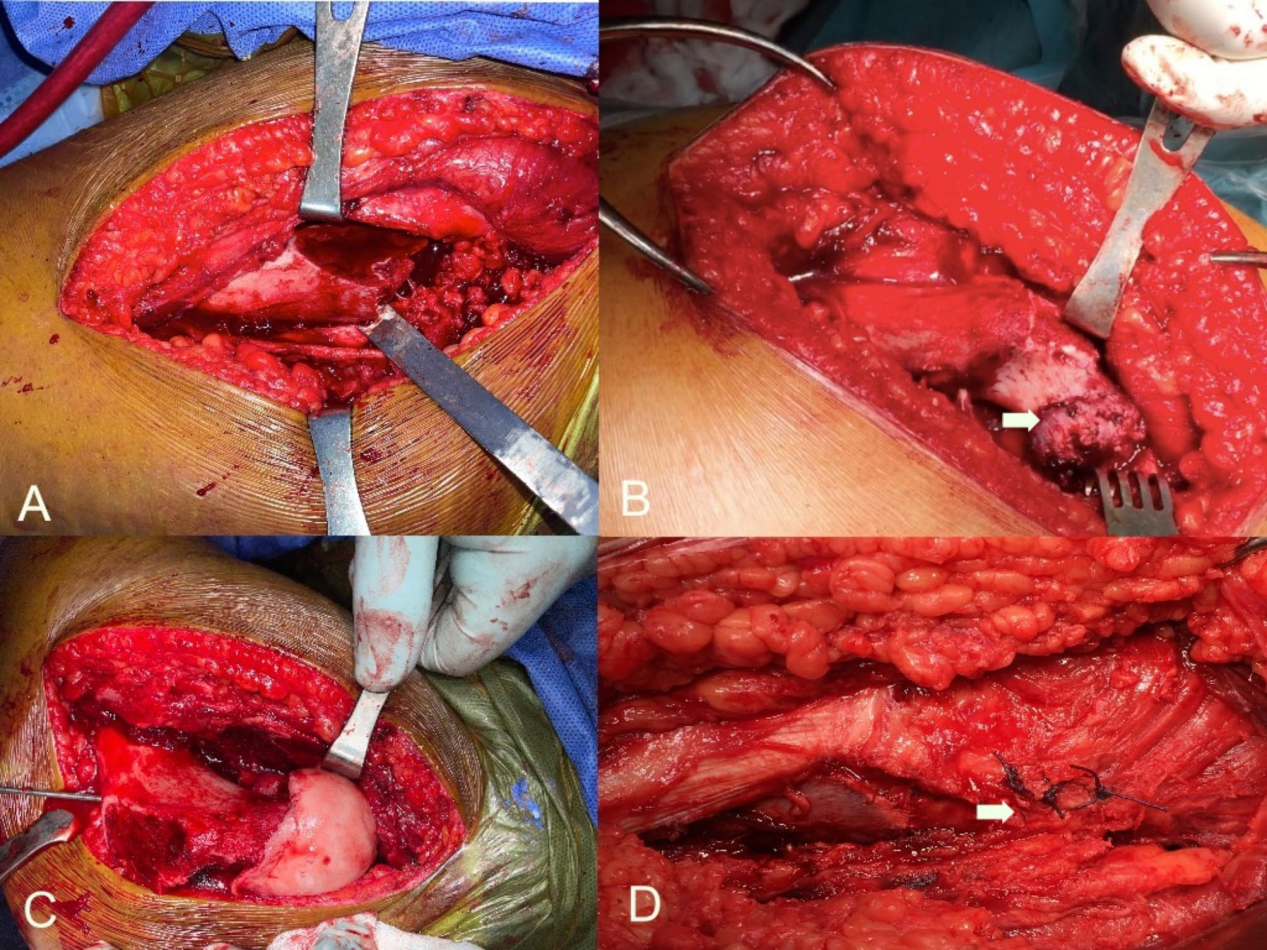
**A**) The bony chip was broken by bending the osteotome posteriorly. **B**) exposure of the posterior neck osteophyte (white arrow) after complete separation of the physis with anterior and posterior retinacular flaps. **C**) Capital realignment and guide wire fixation. **D**) Approximation of the posterior bony chip by Vicryl sutures (white arrow)

The Callus on the posterior aspect of the neck was removed using a straight osteotome. Then, curettage of the proximal stump of the metaphysis was done followed by curettage of the remainder of the growth plate from the inside of the epiphysis. This creates enough neck shortening to allow reduction of the epiphysis without too much tension on the retinacular vessels. After this step, the epiphysis was manually reduced on the metaphysis. The position of the retinacular vessels on the posterolateral aspect of the neck guided the correct rotation of the epiphysis. Further adjustment of the Varus /valgus and ante/retroversion orientation of the epiphysis was performed. When a satisfactory position was obtained, temporary fixation using threaded K-wire in a retrograde fashion was performed. (Fig. 3)

The head was then relocated. Vascularity was rechecked, and an image intensifier was used to check the wire position. When a satisfactory wire position was obtained, another guide wire was inserted to increase fixation of the epiphysis and to avoid rotation of the epiphysis during drilling and screw insertion.

A 7.3 cannulated screw was drilled and inserted over the first guide wire. After checking the final screw position, the hip was tested for stability, and then an approximation of the periosteal sleeve and capsule with loose sutures was made. Trochanteric fixation was done using two 4.5 mm cortical screws. The tiny chip of the stable trochanter released with the posterolateral retinacular flap was approximated to the trochanteric fragment using absorbable sutures.

Weight-bearing and active abduction were restricted for six weeks. Then, gradual weight bearing and active abduction were allowed until 12 weeks. Afterwards, complete activities were permitted.

**Fig. 4:**
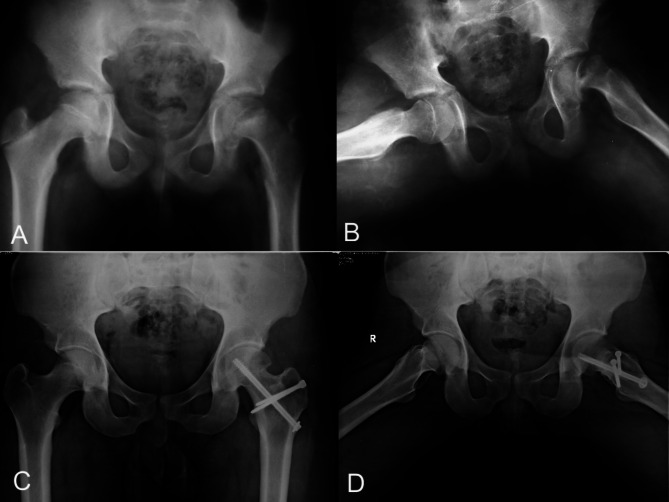
**A**) and **B**): Preoperative radiographs of a 14-year-old male patient with acute on top of chronic SCFE. **C**) and **D**) Final follow-up x-rays done ten years after the surgery show no evidence of AVN or hip osteoarthritis with complete healing of the osteotomy site

**Fig. 5:**
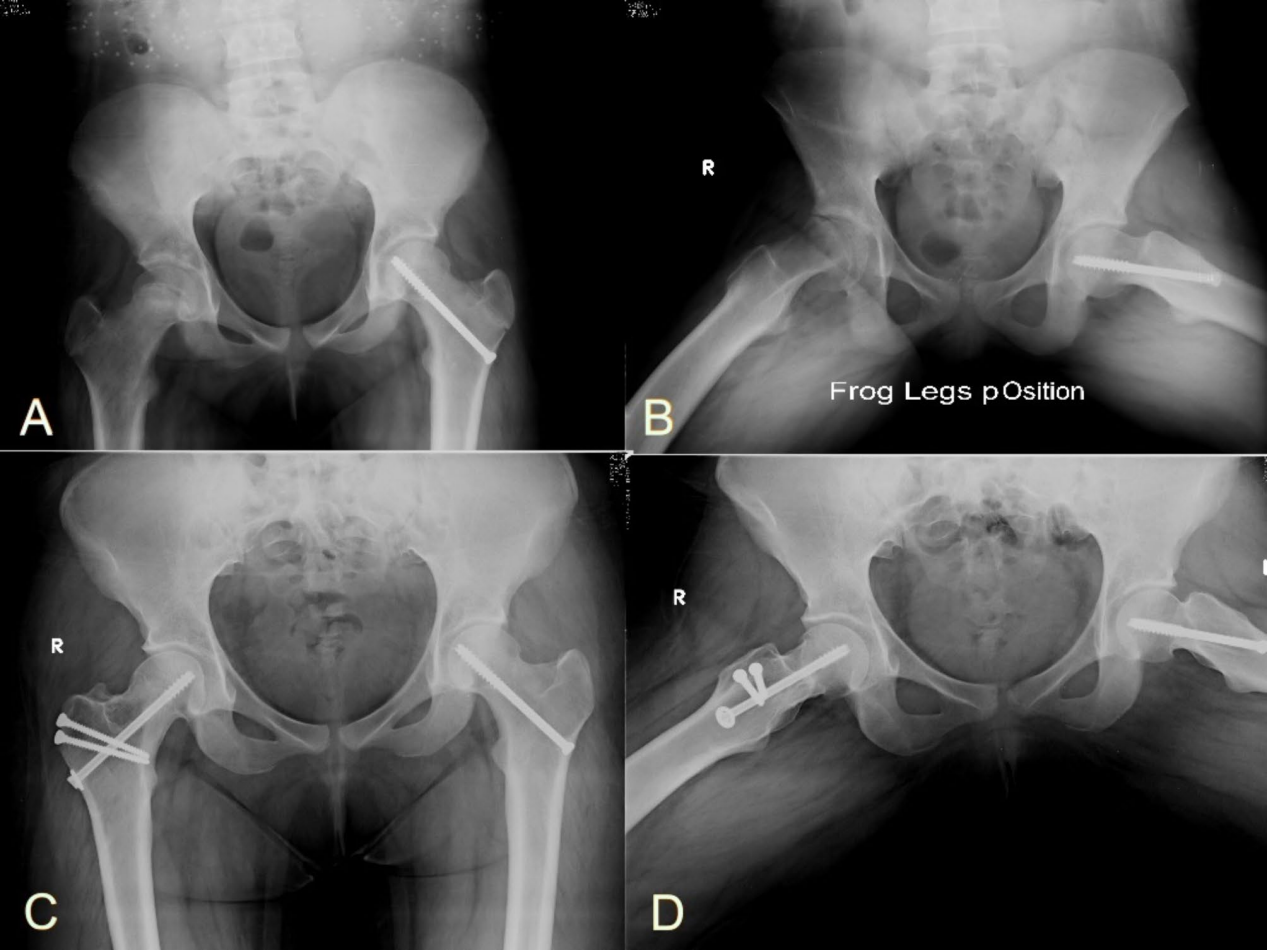
**A**) and **B**): Preoperative radiographs of a 13-year-old female patient with chronic stable SCFE. **C**) and **D**) Final follow-up x-rays done seven years after the surgery show no evidence of AVN or hip osteoarthritis with complete healing of the osteotomy site

**Fig. 6:**
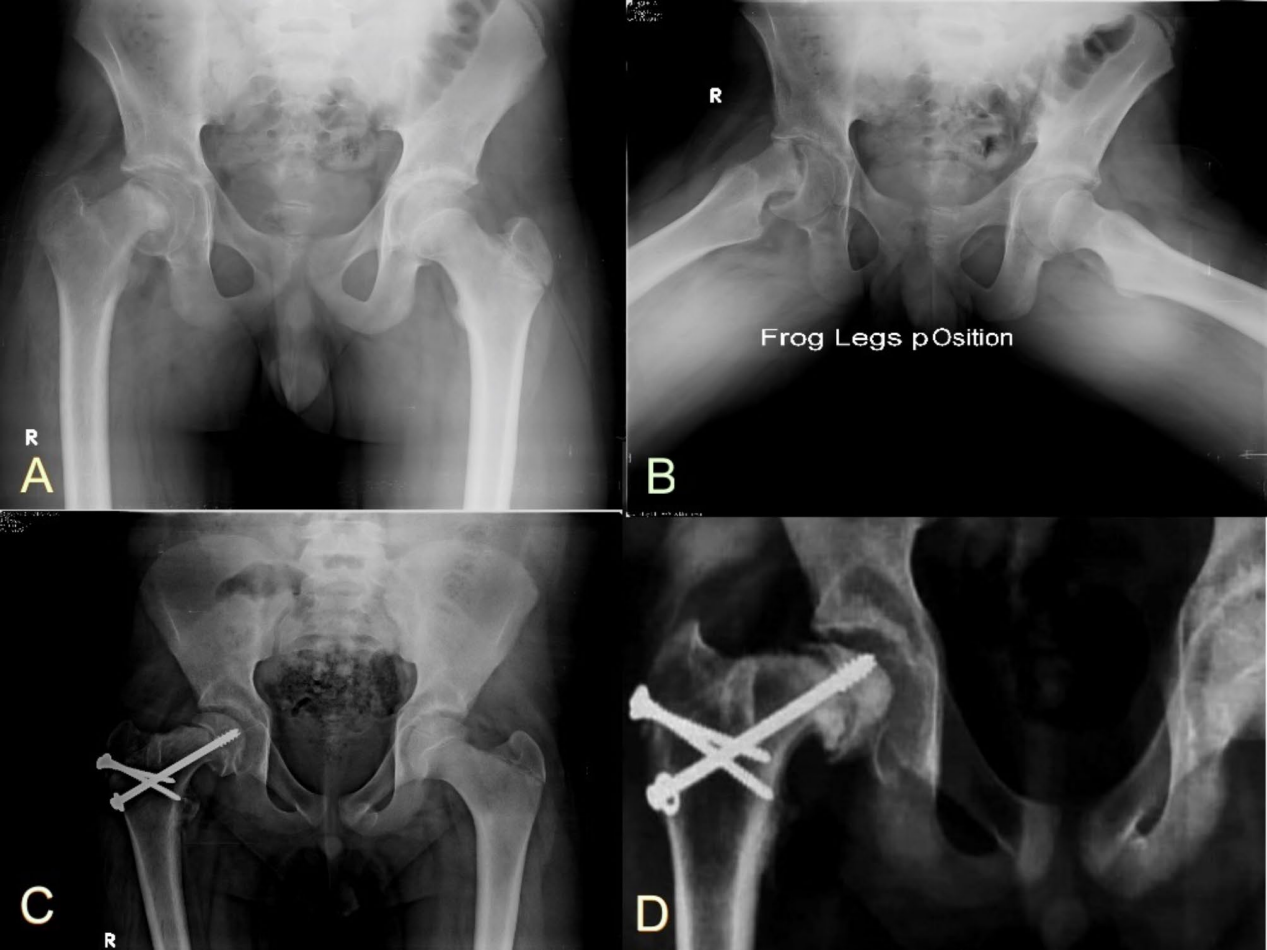
**A**) and **B**): Preoperative radiographs of a 12-year-old male patient who presented with acute on top of chronic SCFE. **C**) An immediate postoperative x-ray showed a varus reduction of the physis. **D**) A follow-up x-ray at ten months showed the development of AVN

Preoperative clinical evaluation included stability according to the Loder classification [9], chronicity according to the Fahey and O’Brien classification [10], the Harris Hip Score (HHS) [11], and the Western Ontario and McMaster Universities osteoarthritis index (WOMAC) [12] score. Post-operative clinical assessment was scheduled for six weeks, 3, 6, and 12 months and annually after that. At the last follow-up, the functional evaluation included a range of motion (ROM), clinical scores, Drehmann`s sign [13], and the FADIR (Flexion Abduction internal rotation) test [14]. The preoperative radiological assessment included the slip angle on the frog-leg or cross-table lateral x-ray. In cases with a doubtful state of the physis, a CT scan was done to confirm the presence of an open physis. At the final follow-up x-rays, the following parameters were measured: the slip angle [1], alpha angle [15], grading of OA according to Tonnis classification and heterotopic ossification (HO) according to Broker et al. [16] (see Fig. 6).

### Statistical analysis

Descriptive statistics were reported for all variables. Chi-squared and two-sample t-tests were used to compare categorical and metric data. The level of significance was set at *p* < 0.05. All analyses were performed using SPSS^®^ software version 27.

## Results

### Patient demographics and baseline characteristics (Table 1)

**Table 1:** Showing the patients` demographics and baseline characteristics

	Number (percent)
Sex	
Male	29 (80.6%)
Female	7 (19.4%)
Side	
Right	14 (38.9%)
Left	22 (61.1%)
Stability (Loder classification)	
Unstable	12 (33.3%)
Stable	24 (66.7%)
Chronicity (Fahey classification)	
Acute	2 (5.6%)
Acute on top of chronic	22 (61.1%)
Chronic	12 (33.3%)
Severity (Southwick classification)	
Moderate	10 (27.8%)
Severe	26 (72.2%)

The study included 36 patients (29 males and 7 females) with an average age of 14 ± 1.19 years and a mean follow-up period of 49.28 months ± 23.10 (range 24–118 months).

### Operative data

The average operative time was 115.44 min ± 21.72, and the intra-operative blood loss averaged 581.03 ccs ± 173.42. Areas of cartilage roughening, and delamination were found in 2 hips (5.5%) and partial labral injuries that needed only debridement were found in 4 hips (11.1%). Partially threaded cannulated screws, 7.3 mm in diameter, were the preferred method of fixation in all cases except only one case (2.8%) that was fixed by 2-mm threaded K-wires.

Only one intraoperative bleeding test was negative (2.8%). Regarding intra-operative physeal stability, 16 hips (44.4%) were unstable, and 20 (55.6%) were stable. Four clinically stable SCFE were found to be unstable intraoperatively. No patients with clinically unstable SCFE were found to be stable at the time of open procedure.

### Clinical and radiographic outcomes (Tables 2 and 3)

**Table 2:** Showing the clinical outcome pre-operatively and at the final follow-up

	Pre-operative	Final Follow-up	*p*-value
HHS (31 hips)	55.81 ± 5.50	89.65 ± 5.27 Excellent 19 (52.77%) Good 12 (33.33%) Fair 0 (0.0%) Poor 0 (0.0%)	< 0.001
WOMAC score (31 hips) Pain Stiffness Function	86.50 ± 3.78 17.31 ± 1.6 6.64 ± 0.89 62.29 ± 3.73	3.15 ± 2.22 0.86 ± 1.57 0.69 ± 1.24 2.58 ± 1.49	< 0.001 < 0.001 < 0.001 < 0.001

**Table 3:** Radiographic outcomes

	Preop	postop	Last follow up	*p*-value
Slip angle (36 hips)	64.56 º±9.50	17.78 º ±5.85	NA	< 0.001
Alpha angle (31 hips)	NA	NA	56.97 º ±5.10	
OA (Tonnis classification) (31 hips)	NA	NA	0: 29 (93.6) 1: 2 (6.4%) 2: 0 (0.0%) 3: 0(0.0%)	

(NA) not available

Five cases developed AVN at a mean of 7.8 months after the operation. For the remaining 31 hips, the HHS and the WOMAC scores showed statistically significant improvement at the final follow-up with a mean of 89.65 ± 5.27 and 3.15 ± 2.22 respectively. Four patients showed a positive Drehmann sign (11.1%), and three (8.3%) had a positive FADIR test at the last follow-up visit. The average final ROM was 109.68º ±11.40 flexion, 17.90 º±5.13 adduction, 40.65º ±4.61 abduction, 45º±4.08 external rotation and 39.19º ±5.64 internal rotation. For all 36 hips, the mean postoperative slip angle was 17.78 º ±5.85 compared to preoperative slip angle of 64.56 º±9.50 (p-value < 0.001). For the remaining 31 hips, the final alpha angle averaged 56.97º ± 5.10. No patient developed heterotopic ossification. No signs of OA were observed in 29 hips (see Figs. 4 and 5). Only 2 hips showed grade 1 OA according to Tönnis classification.

### Post-operative complications

Five patients developed AVN of the femoral head (13.9%), out of which 4 cases had a positive bleeding test intraoperatively (Figure 6). Four AVN cases occurred in the first half of cases compared to one in the second half (*p* = 0.338).

Looking at the risk factors for the development of AVN, none of the following factors showed statistical significance pre-operative (*p* = 0.733) or intra-operative stability (*p* = 0.829), severity of slip (*p* = 0.104) or chronicity (*p* = 0.818).

One patient developed chondrolysis (2.8%), and one patient (2.8%) had hardware penetration (K-wire) into the hip, which was surgically removed.

## Discussion

The results of this case series demonstrate that anatomic reduction with restoration of proximal femoral anatomy is possible with the modified Dunn procedure for moderate and severe slips. This concords with studies reporting good clinical and functional outcomes for moderate and severe SCFE.

The mean postoperative slip angle was 17.78 º ±5.85 as opposed to 64.56 º±9.50 preoperatively, a statistically significant improvement. The residual malalignment in some cases may account for the positive Drehmann sign and the FADIR test in a small number of cases at the latest follow up visit. The preoperative labral and cartilage injuries documented during the procedure could have resulted in the early osteoarthritic changes (Tönnis type I) changes seen in two hips at latest follow up. It is expected that some of these visible changes in addition to other invisible/subclinical changes could result in osteoarthritis in a larger number of hips with further follow up. Their relation to osteoarthritis in cases with SCFE cannot be fully stated due to the small number of cases of osteoarthritis in this series with a mean follow up of only 49 months.

When it comes to the incidence of AVN, we reported 5 cases of AVN (13.8%). The reported incidence of AVN in the literature has been highly variable. Results of the Bernese experience showed 0% AVN [5, 17]. However, higher rates were reported in many other studies [18–20]. Most published case series reported rates between 4% and 19.7% [21–25]. There was a tendency for lower rates of AVN in this study further along the learning curve, although of no statistical significance, a finding reported by many authors [18, 19, 26, 27].

Conversely, Novais et al. [28] found no effect of a learning curve on the rate of AVN. The technical complexity of the procedure can explain this. To precisely calculate the rate of AVN, it should be performed by one or two surgeons at each institution after having previous training. Nevertheless, the number of cases should be large enough to compensate for that steep learning curve. To mitigate the effect of the steep learning curve, surgical training supervised by surgeons experienced in the modified Dunn procedure is of utmost importance. The use of video recordings of entire procedures for later review by the senior and assistant surgeons as well as the trainees, linking them to clinical findings during follow up of the cases may be of help especially in low volume centers. Real time or periodic telecommunication with other centers and the exchange of remarks could further augment the learning capacity in low volume centers. Souder et al. [27] suggested focusing the surgical experience of the modified Dunn on one (or at most a few) surgeon(s) in each hospital should give the best chance for better results. Based on their results, Upasani et al. [19] modified their practice and suggested that a high-volume surgeon must be present during each modified Dunn procedure. This in our opinion poses great difficulty in surgical settings where an experienced surgeon is not available to perform such a demanding procedure in considerable volume. This is a challenge for low-resource areas where SCFE slip angles could still be presenting in moderate and severe degrees, which affects the quality of the management of these young adolescents and predisposes them to early osteoarthritis caused by impingement or indeed by AVN.

We found no significant difference in the rate of AVN between stable and unstable SCFE, which is considered a controversial aspect of SCFE management. Davis et al. [22] compared stable to unstable SCFE, reported a higher incidence of AVN in stable SCFE and recommended against using the modified Dunn procedure in stable SCFE. Similarly, Souder et al. [27] compared modified Dunn and in situ pinning in stable and unstable SCFE. In stable SCFE, there was a significantly higher rate of AVN when using the modified Dunn procedure. In unstable SCFE, there was no significant difference in the rate of AVN.

Similarly, Siroklak [29]reported a high rate of AVN in stable SCFE managed with modified Dunn and advised against its use in severe stable SCFE. Conversely, other authors reported a higher incidence of AVN in unstable SCFE [30–32]. Using anterior subcapital shortening osteotomy in sever SCFE, Mallet el al [33] emphasized that the main risk factor for developing AVN is the unstable nature of SCFE and not the surgeon’s experience.

The minor modification of the apophyseal osteotomy was resorted to in almost all cases in this series. Hence, a comparison to the original technique as a cause for AVN was not possible. A randomized controlled study is needed to establish any risk vs. protective effect of such a modification for the development of AVN.

Hip instability is another devastating complication after the modified Dunn procedure [19, 34, 35]. Upsani et al. [34] reported a 4% incidence of hip instability after modified Dunn procedure for severe chronic SCFE. Chronic external rotation contracture, excessive neck shortening, and valgus reduction are possible factors. However, in our series, we had no cases of hip instability.

Another controversial aspect is comparing the results to in situ pinning, especially in severely stable cases. Novais et al. [28] compared the modified Dunn procedure and in situ pinning for severe stable SCFE. At the short-term follow-up, the modified Dunn procedure resulted in better deformity correction, less hip pain and better ROM than in situ pinning. Regarding complications, there was a relatively lower complication and reoperation rate compared to in situ pinning. However, they emphasised that these results were only for experienced surgeons. Unlike inadvertent reduction and percutaneous pinning for unstable SCFE, Novias et al. [28] concluded that the modified Dunn procedure provided better clinical and radiographic outcomes with a similar proportion of osteonecrosis and unplanned re-operations. Nectoux et al. [36], in a multicenter retrospective study of 222 hips managed with in situ fixation and followed-up for a minimum of 10 years. They concluded that in situ fixation led to impingement in moderate to severe initial slip displacement. The threshold for in-situ fixation should be 35 º slip angle, beyond that other options should be considered.

On the other hand, Trisolino et al. [37] reported similar findings regarding better deformity correction and lower early reoperation rate; the rate of AVN was higher in the modified Dunn group compared to in situ pinning for severe stable SCFE. Comparing the modified Dunn procedure to other osteotomies, Sikora-Clark et al. [29] advised against using the modified Dunn procedure in stable SCFE patients and favoured performing Imhauser osteotomy based on their rates of AVN. Fournuier et al. [38] compared Anterior cuneiform osteotomy with modified Dunn procedure in unstable sever slip. Although clinical and radiological outcome were similar, Cuneiform osteotomy reported less AVN rate than Dunn. Table 4 summarizes the results and complications of most of published studies using the modified Dunn procedure.

**Table 4:** Summary of the results of modified Dunn procedure in different studies

Article	Type of study	Level of evidence	Number of hips	Follow-up period (mean ± SD or range)	Stable Vs unstable SCFE	Affected side Rt/Lt	Age (Mean ± SD or range)	Sex Male/Female	Number of cases with AVN (stable/ unstable SCFE)	Other complications	Conclusions/recommendations
Abdelazeem, 2016 [8]	Prospective Case series	IV	32	31months (12–40)	32/0	21/11	14.3 ± 1.8 years	26/5	1/0	NR	Modified Dunn Procedure is a safe treatment option in stable SCFE with high slip angle
Agashe 2021 [30]	Retrospective case series	IV	30	25.36 months (13–60)	19/11	16/14	13.05 ± 1.41	25/5	0/2	1 Hip subluxation	Modified Dunn procedure is the first treatment option for moderate and severe forms of SCFE
Alves, 2012 [26]	Retrospective case series	IV	12	NR	0/12	NR	12.15 years	6/6	0/6	NR	Modified Dunn Carries a higher risk for AVN development compared to closed reduction and percutaneous pinning
Birke 2021 [21]	Retrospective case series	IV	178	2.7 years (1–9.2)	107/71	78/94	13.5 years (9.5–17.5)	96/76	5/15	7 Hip dislocations 2 FAI 4 peroneal nerve palsy 1 DVT 1 sciatic neuropathy 4 superficial wound infection	They recommended using the Modified Dunn procedure in stable SCFE combined with intra-operative monitoring. It provided equivocal results regarding AVN development in unstable cases.
Davis 2019 [22]	Retrospective case series	IV	48	27.9 months	17/31	NR	12.5 years ± 13.8	NR	5/2	3 hip subluxation or dislocation 4 heterotopic ossifications 4 hardware failure	The procedure carries a higher risk for AVN and hip instability in patients with stable SCFE with inferior restoration of the proximal femoral anatomic parameters. It should be used with caution in patients with chronic, stable SCFE
Fournier, 2022 [38]	Retrospective case series	IV	41	2–4 years	0/41	NR	11.5–14.9 years	17/24	0/8	2 chondrolysis 2 FAI	Cuneiform osteotomy has equal result to modified Dunn in the treatment of severe unstable SCFE
Gabana, 2022 [20]	Retrospective case series	IV	19	NR	NR	12/7	11.9 years ± 1.8	8/11	7 (NR)	1 hardware failure 6 Secondary FAI 1 hip instability	The procedure carries a high risk for the development of AVN in cases with severe SCFE. No specific risk factor was associated with the development of AVN.
Galletta, 2021 [23]	Retrospective cohort	III	81	5.7 years ± 3.3	NR	35/46	13.6 years ± 1.9	59/17	16 (NR)	2 hardware failure 9 conversions to THA 2 trochanteric non-union	Modified Dunn procedure carries an equal risk of AVN compared to in situ pinning in moderate and severe stable slips.
Jackson, 2018 [40]	prospective case series	IV	9	22 months	0/9	NR	12.2 years (11 − 9)	5/4	2(unstable)	(NR)	Super-selective medial circumflex femoral artery angiography was performed pre-and post-operatively to assess femoral heal perfusion in cases with unstable SCFE, supplemented by an intra-operative evaluation of perfusion using an intracranial pressure Monitor. There was no procedure-induced loss of perfusion with six cases having pre-operative blood flow to the femoral head.
Lerch, 2019 [14]	Retrospective case series	IV	46	9 months (4–20)	70/30	20/80	13 years ± 2	65/35	2 (NR)	1 hardware failure 3 Secondary FAI	The procedure carries an acceptable complication rate for cases with severe SCFE except for the development of FAI which might need further surgical interventions
Leunig, 2007 [5]	Retrospective case series	IV	30	55 months (24–96(	NR	7/23	13 years (10–17)	NR	0	2 hardware failure	The original case series described the procedure. They followed the patient for an average of 55 months, with no evidence of AVN.
Madan, 2013 [41]	Prospective case series	IV	28	38.6 months (24 to 84)	11/17	NR	12.9 years (10 to 20)	14/14	0/4	No other complications	In this early case series, the authors had only 2 cases of AVN. No other complications were reported, and the procedure was considered as a safe option for all types of SCFE.
Masquijo 2019 [31]	Retrospective Case series	IV	21	40.4 months (12–84)	15/6	13/8	12 years (10–16)	10/10	2/8	1 superficial infection 1 conversion to THA	This was a multicenter study where again the authors acknowledged the technical complexity and high learning curve of the procedure, which accounted for their high complication rate.
Novais, 2015 [39]	Retrospective cohort study	III	15	2.5 years (1–6)	15/0	NR	NR	NR	1/0	1 hardware failure 1 conversion to THA	The procedure has a similar complication rate and better femoral morphologic features compared to in situ pinning in stable severe SCFE.
Novais, 2019 [28]	Retrospective cohort study	III	27	2.4 years (1.8–3.1)	0/27	NR	12.6 years (11.8–13.5)	15/12	7	1 hardware failure 4 conversions to THA	Comparing the procedure to in situ pinning for cases of unstable SCFE, it shows better clinical and radiographic outcomes with a similar proportion of osteonecrosis and unplanned re-operations
Persinger, 2018 [24]	Retrospective case series	IV	31	29.3 months (12–82)	0/31	11/20	12.37 (8.75–14.8)	15/15	2	1 hardware failure	The study was a single surgeon study, where 31 cases of unstable SCFE were recruited. It concluded that the modified Dunn procedure was a safe and effective procedure for unstable SCFE with acceptable complication rate.
Sankar, 2013 [18]	Retrospective case series	IV	27	22.3 months ± 12.48	0/27	9/18	12.6 years (9.7–16)	17/10	7	4 hardware failure 1 conversion to THA	This was the first reported multicenter study which included also included patients with unstable SCFE only. It again demonstrated the superiority of the procedure in restoring the anatomical parameters, but with a considerable risk of complications including AVN (26%)
Sikora-Klak, 2019 [29]	Retrospective cohort	III	14	29 months ± 8.9	14/0	NR	13.1 years ± 1.9	8/6	4	2 hardware failure 2 conversions to THA	The authors of this study compared two procedures: the modified Dunn procedure and the tri-planar femoral osteotomy. They advised against the use of the modified Dunn procedure in stable cases as all their 4 cases who developed AVN were in the modified Dunn group. In contrast, no patients in the tri-planar osteotomy group developed AVN, despite its technical difficulty.
Trisolino, 2018 [37]	Retrospective case series	IV	29	4.3 years ± 2.6	29/0	12/17	13.9 years ± 2.3	22/7	3	2 conversions to THA	The study compared the modified Dunn procedure to in situ fixation only in severe SCFE. Three patients developed AVN, while none developed AVN in the in-situ group. This raised suspicions against the procedure’s safety in severe SCFE; however, overall, both groups had comparable re-operation rates for different reasons.
Upasani, 2014 [19]	Retrospective case series	IV	43	2.6 years	17/26	10/7	12 years ± 1.7	18/25	10 (NR)	NR	The study was a single-centre experience that involved consecutive recruitment of patients with all forms of SCFE. The study revealed an inverse relationship between surgeon volume and outcomes. So, they modified their practice by having an experienced surgeon present at each procedure. Additionally, only patients with acute severe (> 50 degrees) epiphyseal displacement with mild chronic remodelling of the metaphysis who can be treated within 24 h of the slip were offered the modified Dunn technique.
Upasani, 2017 [34]	Retrospective case series	IV	17	2 years	10/7	NR	13 years	10/7	14 (NR)	3 conversions to THA 8 hip instability	The main aim of the study was to investigate iatrogenic hip instability after the procedure. Seventeen patients developed post-operative anterior hip instability. The researchers proposed that the patients might benefit from a period of bracing using either an abduction brace or broomstick cast to reduce the chances of future hip instability.
Ziebarth 2009 [17]	Retrospective case series	IV	40	3.8 years (1–8)	28/12	12/28	12.6 years (9–18)	17/23	0	4 Heterotopic ossifications 1 FAI 3 hardware failure	In this case series, no patient developed AVN. They supported the procedure for moderate and severe forms of SCFE.
Our Study	**Prospective case series**	**IV**	**36**	**49.28 months ± 23.10 (range 24–118 months).**	**24/12**	**14/22**	**14 ± 1.19**	**29/7**	**3/2**	**1 chondrolysis (2.8%)**, **one (2.8%) had hardware penetration (K-wire) into the hip, which was surgically removed**	**the modified Dunn procedure is a safe option for treating moderate to severe SCFE. It provides superior femoral head realignment and patient-reported outcomes. It can be safely performed by experienced surgeons with accepted complication rates, including AVN.**

SCFE: Slipped Capital Femoral epiphysis, AVN: Avascular Necrosis, NR: Not Reported, FAI: Femoroacetabular Impingement, DVT: Deep Venous Thrombosis, THA: Total Hip Arthroplasty

Intraoperative femoral head bleeding or intracranial pressure measurement (ICP) effectively identifies patients at risk of developing AVN [39]. It is a safe, real-time tool for intraoperative assessment of the femoral head perfusion [40]. Madan et al. [41] demonstrated high femoral head active bleeding specificity in predicting AVN. None of the four cases that developed AVN in their series had active bleeding from the femoral head before dislocation or after reduction. Similarly, Jackson et al. [40]. demonstrated that the absence of blood flow by ICP monitoring strongly predicts AVN. Contradictory to these results, Upsani et al. [19] found that intraoperative femoral head blood monitoring did not correlate with outcome. In their series, five patients had no intraoperative bleeding from the femoral head. However, only one of these five patients developed AVN. However, nine patients developed AVN who either had bleeding from the femoral head or triphasic waveforms with ICP monitoring of femoral head perfusion. Sankar et al. [18] reported four patients with AVN despite confirmed blood flow after fixation. Our results align with those of Upsani et al. and Sanker et al., where 4 cases developed AVN despite positive bleeding test intraoperatively. ICP monitoring could improve the outcome by optimising femoral head perfusion throughout the surgical procedure by alerting the surgeon to decrease femoral head perfusion after specific steps that stretch the retinaculum and decrease femoral head perfusion [19, 21, 40].

The main limitation of our study is the absence of a control group to compare the procedure’s effectiveness to, but the lack of cases can explain this. A larger number of cases or a longer follow up of the existing series might result in correction of a possible type II error and increase the power of this study in order to establish causality as regards a devastating complication like AVN. However, our findings can be considered robust evidence because they are a prospective case series compared to other published studies, which were retrospective. Additionally, having all the cases performed by the same surgeon enabled us to judge and estimate the learning curve of the procedure accurately. Consistency in performing the steps of the surgical technique, the vigilance and meticulous assistance by the helping team of other surgeons as well as accurate documentation of intraoperative findings are key to building up experience and climbing up the steep learning curve.

## Conclusion

Based on our findings, the modified Dunn procedure is a safe option for treating moderate to severe SCFE. It provides superior femoral head realignment and patient-reported outcomes. It can be safely performed by experienced surgeons with accepted complication rates, including AVN.

## Data Availability

No datasets were generated or analysed during the current study.
